# Applying a Commercial Determinants of Health Lens to Understand, Expose and Counter Industry Co-option, Appeasement and Partnership: Comment on "‘Part of the Solution’: Food Corporation Strategies for Regulatory Capture and Legitimacy"

**DOI:** 10.34172/ijhpm.2022.7371

**Published:** 2022-07-11

**Authors:** Eric Crosbie, Angela Carriedo

**Affiliations:** ^1^School of Public Health, University of Nevada Reno, Reno, NV, USA.; ^2^Ozmen Institute for Global Studies, University of Nevada Reno, Reno, NV, USA.; ^3^World Public Health Nutrition Association, London, UK.; ^4^Department of Health, University of Bath, Bath, UK.

**Keywords:** Commercial Determinants, Health Harming Industries, Public Private Partnerships, Conflict of Interest

## Abstract

Lacy-Nichols and Williams’ examination of the food industry illustrates how it altered its approach from mostly oppositional to regulation to one of appeasement and co-option. This reflection builds upon this by using a commercial determinants of health (CDoH) lens to understand, expose and counter industry co-option, appeasement and partnership strategies that impact public health. Lessons learned from tobacco reveal how tobacco companies maintained public credibility by recruiting scientists to produce industry biased data, co-opting public health groups, gaining access to policy elites and sitting on important government regulatory bodies. Potential counter solutions to food industry appeasement and co-option include (i) understanding corporate actions of health harming industries, (ii) applying mechanisms to minimize industry engagement, (iii) dissecting industry relationship building, and (iv) exposing the negative effects of public private partnerships (PPPs). Such counter-solutions might help to neutralise harmful industry practices, products and policies which currently threaten to undermine healthy food policies.

Lacy-Nichols and Williams’ examination of the evolving change of the food industry over the last couple of decades provides important implications for public health and beyond. This insightful piece documents how the food industry altered its approach from mostly oppositional and sometimes hostile to regulation to one of appeasement and co-option.^1^ Characterizing this approach as ‘part of the solution’ Lacy-Nichols and Williams illustrate how this strategy emerged and diffused through the food industry in the context of growing market and regulatory threats to the industry. In particular, they detail how this ‘part of the solution’ strategy can be characterized by agility and responsiveness as seen through three key pillars, (1) regulatory response and capture, (2) relationship building, and (3) new market strategies. In this reflection, we discuss the role of corporate credibility in social norms, driven by the three key pillars of strategies and responses identified by the authors. We build upon this analysis by emphasizing the importance of using a commercial determinants of health (CDoH) lens to help explain the vector-host-disease epidemiology causal pathway of diseases.^2^ In particular, we illustrate lessons learned from tobacco control as well as some key examples that have emerged in food and nutrition as potential solutions to counter these corporate actions and strategies.

## Applying a CDoH Approach to Understand Disease Pathways

During the 20th century substantial progress was made in controlling and preventing infectious diseases (eg, malaria, tuberculosis, and HIV/AIDS). In contrast, in the 21st century we are currently witnessing a dramatic increase in non-communicable diseases (NCDs) (eg, cancer, heart disease, diabetes), which account for approximately 41 million deaths per year, representing 71% of annual global deaths, of which 85% occur in low- and middle-income countries.^3^ Two-thirds of all NCD deaths are related tobacco use, alcohol misuse, poor diet and physical inactivity.^3^

While biological, behavioral, and social elements are all determinants of NCDs, over the last decade a new wave of research and concentration has begun to systematically focus on corporate and commercial factors that negatively impact health. CDoH, which are strategies and approaches used by businesses and corporations to promote products and choices that are harmful to health,^4^ encompass the three pillars the authors explored (1) regulatory response and capture, (2) relationship building, and (3) new market strategies, among others. In doing so, emerging CDoH frameworks recognize *proximal* risk factor determinants (direct or downstream) impacts on disease/injury and death but shift focus towards more *distal* causes (upstream socioeconomic and environmental determinants) that shape proximal risk factors.^5^ In particular, health harming industries (such as tobacco, alcohol, ultra-processed food and beverage, pharmaceutical and fossil fuels) act as commercial vectors of disease that infect populations (host) through marketing practices, capturing institutions, delaying policy implementation, among others (environment) that encourage the consumption of unhealthy commodities (agent). Thus, further application of a CDoH approach can help guide our observations and research to understand the causal pathways of injury/disease and death and provide solutions to such identified problems related to commercial actions, strategies and approaches.

## Understanding and Exposing Commercial Vectors: Lessons From Tobacco Control

For decades the tobacco industry acted as a legitimate stakeholder until the 1990s when the public discovered that the companies had lied about the addictive nature of nicotine.^6^ Through lawsuits in the United States, previously secret internal tobacco industry documents were made publicly available, which were digitalized in the UCSF (University of California, San Francisco) Industry Documents Library among other public domains. These documents severely hurt the industry’s credibility as they provided a firsthand look into understanding the industry’s internal planning, political practices and marketing strategies. Researchers were able to get behind the veil and essentially study the corporate vector of disease (tobacco industry) and expose its deceptive tactics to undermine the negative impact of tobacco on health.^7^ These efforts led to important published research that revealed how tobacco companies for decades maintained public credibility by recruiting scientists to produce industry biased data to downplay the effects of smoking and secondhand smoke, publishing favorable industry positions in prestigious academic journals, co-opting and dividing public health groups, gaining access to policy elites and sitting on important membership boards and government regulatory bodies.^8,9^

Exposing the tobacco industry vector also helped lead to the adoption of the first and only global health treaty, the World Health Organization (WHO) Framework Convention on Tobacco Control (FCTC), which recommends a series of supply and demand-side measures to reduce tobacco consumption globally. More importantly, the FCTC established Article 5.3, which essentially prevents tobacco companies from participating in government meetings and decision-making policy processes (aka part of the solution). The implementation of FCTC Article 5.3 guidelines has led to important fundamental shifts in minimizing tobacco industry executives and lobbyists from interacting with government officials and influencing policy decisions.^10^ Yet, scholars have found that implementing FCTC Article 5.3 has not been easy as further mechanisms are needed to apply to specific contexts and deal with issues such as existing public-private partnerships. Additionally, the FCTC is still far from being implemented entirely in some low- and middle-income countries due to limited understanding and engagement beyond health agencies, as some government agencies (eg, trade), continue to work closely with the tobacco industry as close allies.^11^

Given these challenges civil society groups could examine FCTC Article 5.3 as a policy instrument to highlight success in establishing awareness and support for fundamental norm change related to industry conflicts of interest (COI).^10^ These groups could further encourage policy-makers to establish whole-of-government (multiple government departments) cohesive policies which could help minimize inter-sectoral conflict and align in reducing health harming industry influence.^10^

## Solutions to Counter Industry Co-option, Appeasement and Partnership

As with tobacco, there are important ways to counter the credibility and power of food, beverage and agribusiness corporate actors in public health policy-making. We propose some of these solutions, which include (*i*) understanding and exposing the corporate actions of health harming industries, (*ii*) developing and applying mechanisms to minimize engagement with health harming industries, (*iii*) revealing the networks and relationship building among public and private actors, and (*iv*) exposing the negative effects of public private partnerships (PPPs) for policy implementation.

###  Understanding and Exposing the Corporate Actions of Health Harming Industries

One way to investigate corporate actions of health harming industries is by exploring internal industry documents to understand and expose their ‘part of the solution’ narratives and actions. This includes the UCSF Industry Documents Library, which initially began collecting and digitally archiving only tobacco industry documents but in the last decade has expanded to include internal industry document collections for chemical, drug, food and fossil fuel industries. Notable findings from these industry documents have already exposed the food industry’s targeted marketing of ethnic groups,^12^ and its efforts to control and privatize public water supplies in countries that face water scarcity.^13^ Other notable ‘corporate watch’ programs and databases include Tobacco Tactics, US Right to Know, Project Toxic Docs, Preemption Watch, Open Secrets, Transparency International, among others. Another way to investigate commercial actions, particularly around policy-making is by conducting freedom of information requests to secure government documents that reveal important information related to industry meetings with policy-makers, public comment submissions to committees, and negotiations with trade representatives.^14^ These efforts could help counter the industry’s ability to be highly involved as a key stakeholder in policy design, implementation and evaluation and proactively help expose the long-lasting engagements and normalization of such relationships, which have been a challenging threat to the policy-making space.

###  Developing and Applying Mechanisms to Minimize Engagement With Health Harming Industries 

While there does not exist a global public health treaty for other unhealthy commodities such as the FCTC, important policy developments have occurred that implement some of the elements expressed by FCTC Article 5.3 to restrict and minimize industry involvement in policy design and implementation. The WHO tool to restrict food industry interference, which has been further developed in some regions (eg, the Americas),^15^ provides an opportunity for WHO Member States to evaluate industry behavior before engaging with these actors on policy decisions. Other international examples include reports published by the Organization for Economic Cooperation and Development with assistance tools on how COI are managed and resolved in countries, and the EuroPam, a project initially hosted by the World Bank, that holds European Union country profiles on COI, accountability mechanisms, and enables whistleblowers to report COI in governments. However, to date application of mechanisms to protect the public health policy space from health harming industries are limited, and mainly target the tobacco industry.^16^ Few examples that explicitly limit such relationships exist to date.^16^ As with tobacco, exposing industry practices has led to changes in policies and establishing mechanisms to protect policy-making and research. For example, some universities have changed their COI policies, which have forced some professors to give back their funding and support from the food industry.

###  Revealing Corporate Networks and Relationship Building

Similar to studying other vectors of disease, dissecting the corporate vector from within can provide details on the important role corporate networks and relationship building of health harming industries has in sustaining their credibility and participation. Overcoming industry relationship building is a complex strategy but with the aid of better science around policy networks analysis, and exposure and potential risk associated to those, can support efforts to better scrutinize actors close to the decision-making venues.^17^ For instance, investigation around the connection and revolving doors of politicians and industry, has been effective in further accountability demands of claim-holders.^14,17^

Furthermore, analyzing industry global commodity chains can expose how particular parts of the commodity chain have been exploited by corporate elites.^18^ Surveillance and anger from food producer and peasant groups, key members of food production chains and largely ignored in the political economy of food corporations, have risen up against corporate actions and narratives. For example, generating greater visibility in the media and collective global civil society discussions resulted in a strong movement and opposition against the Food System Summit lead by the United Nations, an initiative to transform the global food system into a more sustainable and equitable one.^19^ The civil society mechanism, representing many claim-holders globally, opposed the initiative, as it was perceived as an industry-coopted one.^19^

###  Exposing the Negative Effects of Public Private Partnerships for Policy Implementation

Another important area to expose is the industry’s usage of PPPs to establish and maintain credibility. PPPs, which are typically collaborations between government agencies and private sector companies to finance, build and operate projects (eg, building a hospital), have increasingly grown due to the demand to find alternatives for financing public programs. Despite industry promises that these programs will help the public, PPPs to date have produced minimal public gains and instead have allowed industries to appeal to resource strapped agencies, establish entrenched cooperative government relations, and ultimately gain credibility to avoid government regulations.^20,21^ Thus, it is important to question these industry entanglements and implement mechanisms that establish transparency guidelines, COI disclosures, and accountability measures to ensure these projects benefit the public rather than corporate interests.^20,21^ While government funding continues to be a constraint in rejecting industry lead PPPs, generating further discussion and accountability measures can help minimize these entanglements that reinforce private interests over public interests such as the UK Responsibility Deal.^22-24^ Weak architecture for the global governance of nutrition and disagreements in the nutrition community on the advisability of engaging the private sector, are challenges faced to resist these engagements in relation to food policy.^25^

## Conclusion

 Adopting a CDoH approach helps us identify health harming industries as corporate vectors of disease and regulate their practices to better address the NCD epidemic. Exposing industry harming practices, products and policies, in combination with advocacy strategies, and government accountability mechanisms, have shown to be antidotes for addressing the vector of disease. Research exploring how these changes are affecting social norms and behaviors positively can provide evidence to further support solutions that minimize industry ‘part of the solution’ narratives and approaches.

## Ethical issues

 Not applicable.

## Competing interests

 Authors declare that they have no competing interests.

## Authors’ contributions

 EC and AC conceptualized the idea and together co-wrote and revised the article.
